# Supplementary material for: CancerInSilico: An R/Bioconductor package for combining mathematical and statistical modeling to simulate time course bulk and single cell gene expression data in cancer
Source: PLoS Comput Biol. 2019 Apr 19;14(4):e1006935. doi: 10.1371/journal.pcbi.1006935 (PMC6504085; doi:10.1371/journal.pcbi.1006935)
Supplement: S1 Text — (PDF) [file pcbi.1006935.s001.pdf]

CancerInSilico Supplemental Material

Introduction

The purpose of this supplement is to clearly describe the underlying machinery of the *CancerInSilico* package. Whereas the main manuscript focused on the high level design of the package and demonstrated some use cases, this document will focus on a detailed explanation of the parameters and models implemented in the package.

The main feature of *CancerInSilico* is the simulation of gene expression data. There are three primary components of this simulation: the cellular growth model, the pathway activity simulation, and the platform specific error model. Each of these components contain distinct parameters that can be controlled by the user, and so understanding how these components work together is critical for assessing parameter sensitivity. Given the number of parameters available to the user, parameter sensitivity is a critical part of any analysis done with *CancerInSilico*.

Cellular Growth Model

General Model Framework

The base component of the gene expression simulation is the cellular growth model. This model serves as a reference point for all downstream data simulations. The package is designed to allow the user to seamlessly switch between many different model implementations. In order to accommodate this, all models must be implemented as part of a hierarchy. This hierarchy describes different levels of a cell simulation so that the common portions can be shared across different implementations. It also means that the parameters for the growth model exist in a hierarchy. We will first address the CellModel parameters which refer the top level parameters common to all model implementations.

**CellModel Parameters**		
**Parameter Name**	**Description**	**Value**
cellTypes	All cell types present in the simulation	list of CellType objects
initialNum	Initial number of cells	integer > 0
cellTypeInitFreq	Frequency of each cell type at the start	vector of weights
boundary	Constrain cells within circular boundary	boolean
density	Initial density of the cells	$0 <$ double < 0.5
syncCycles	Synchronize cell phase across all cells	boolean
runTime	Total number of simulated hours	double > 0
timeIncrement	Amount of time passed during each time step	double > 0
drugs	All drugs present in the simulation	list of Drug objects

CancerInSilico only supports cell-based model implementations, meaning that each cell is modeled individually as part of a larger population. Since the cell is the fundamental unit of the model, it is convenient to define cell types which describe the behavior of a class of cells. In *CancerInSilico*, a CellType is an S4 class [4] in R which details the distribution of growth rates and the size of all cells of that type. When a new cell is born, its expected growth rate is sampled from the distribution provided by its CellType. The expected growth rate is given as a target number of hours for the cell cycle to complete. The actual length of the cell cycle will depend on environmental factors present in the model.

Most of the other top level parameters detail how the growth model is initialized. **initialNum** and **cell-TypeInitFreq** specify the number and types of the initial cells in the simulation. **boundary** allows cells to either grow freely in space or be constrained by a circular boundary. **density** determines how closely packed the cells are within this boundary. **syncCycles** allows the user to have every cell start off at the same point of the cell cycle, otherwise they are seeded at random points throughout the cycle. **runTime** and **timeIncrement** control how long the simulation runs for and how long each update step takes. The time increment is chosen to be sufficiently small so that the simulation has enough granularity to satisfy the model mechanics without breaking any constraints. This value is determined from the other model parameters. Finally, **drugs** allow the user to specify any number of drugs that will take effect at some predetermined time. The effect of each drug is to reduce the expected growth rate of a cell by some amount determined by a user-defined function.

Off-Lattice Model

The parameters general to all simulations do not contain any information about the actual model dynamics. The model dynamics are implemented in the lower levels of the model hierarchy. Before defining the specific model dynamics, it is useful to group models by their geometrical representation of cells, i.e. off-lattice and on-lattice models. This distinction is convenient since models that share the same geometrical representation tend to share many other properties as well. Furthermore, the cell population data structure will be implemented differently depending on the cell geometry, so from a software design perspective this is also a useful distinction. The first model implemented in *CancerInSilico* is an off-lattice model so we will describe that geometry here. This formulation for an off-lattice cell comes directly from the Drasdo and Höhme model [1], but could be used for other off-lattice models as well.

Cells are described as two overlapping circles. During Interphase the distance between the centers is zero, so the cell is just a simple circle. During Mitosis, however, the distance between the centers grows and the entire shape deforms. Once the process of mitosis is complete, two circles of the original cell size are leftover. During Interphase the cell doubles in area, which allows for the entire Mitosis process to happen without changing the total area of the cell. The length of time it takes for the cell to double in area is controlled by the expected growth rate of the cell.

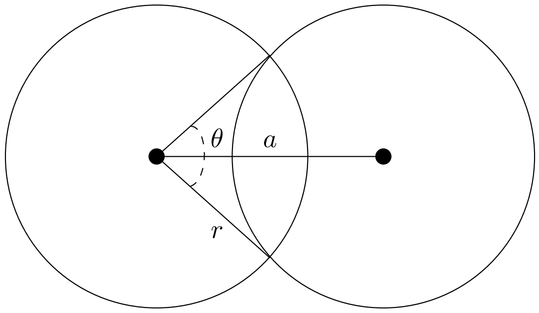

Drasdo-Höhme Model

The geometry of the cells in an off-lattice model provide the core structure, but the updating procedure is responsible for enforcing the desired cell mechanics. The Drasdo-Höhme model outlines an updating procedure which involves several types of possible changes to the cell configuration and a method for accepting or rejecting a proposed change. During Interphase, cells can move and grow. During Mitosis, cells can move, rotate, and progress farther along the division process (separate). The model defines a total energy function on the configuration of all the cells. This energy function is responsible for making the cells adhere to some ideal configuration. Changes that move the cells away from the ideal configuration are more likely to be rejected.

It would be possible to have parameters controlling each possible change to the cell configuration. These parameters, however, would not be inuitive to the user. Drasdo and Höhme present three master parameters which encapsulate the rest of the mechanical parameters in a way that is easier to relate to a physical notion [1].

**Drasdo-Höhme Parameters**		
**Parameter Name**	**Description**	**Value**
n_g	Ratio of growth steps to non-growth steps	integer > 0
δ	Distance for short range interactions	double > 0
ϵ	How strictly the cells conform to ideal configuration	double > 0

The n_g parameter controls how many update steps are done in between growth steps. This essentially allows more time to find an ideal configuration of cells before attempting a growth trial. A high value for this parameter ensures that growth trials are rarely rejected but comes at the expense of computation time. This is important since the actual growth rates should not differ from the expected growth rates unless some other environmental factor is present. The effect of δ is apparent in the total energy function given as:

$$V^{tot} = \sum_{i<j} V_{ij}$$

$$V_{ij} = \begin{cases} \left(\frac{2d_{ij}}{\delta} - 1\right)^2 - 1 & 0 \leq d_{ij} \leq \delta \\ \infty & otherwise \end{cases}$$

d_{ij} = distance between cells i and j.

If two cells are more than δ apart then no interaction will occur. If two cells are within δ then they will never be separated. This feature gives a natural definition of cell neighbors. Once a change to a cell is proposed, it is accepted with probability:

$$P_{accept} = \begin{cases} e^{-\epsilon \Delta V^{tot}} & \Delta V^{tot} \geq 0 \\ 1 & \Delta V^{tot} < 0 \end{cases}$$

This equation makes the effect of ϵ apparent: the larger ϵ is, the smaller the chance of accepting a change that increases the total energy. With this formulation, the Drasdo-Höhme model then ensures that by updating the cells accordingly, the total energy function will be minimized for the given cell population. The total energy function is minimized when $d_{ij} = \delta/2$ for all neighboring cells i, j. This simple energy function enforces cell-cell adhesion and provides the cells enough room to grow [1].

Pathway Activity Simulation

Before the model state can used to generate gene expression data, there has to be a mapping from the model state to the activity in all the genes of interest. This mapping happens through a list of user defined pathways. Each pathway contains a set of genes and a function to determine the activity in those genes given the model state. The pathway activity is a value from 0 to 1, where 0 represents no activity and 1 represents maximum activity. This activity measurement will serve as the basis for generating mean expression levels in the next phase of the simulation.

The user-defined pathways are not so much parameters as they are the mechanism for encoding a biological hypothesis. The purpose of *CancerInSilico* is to test the performance of analysis methods on hypothetical, simulated data sets. By defining how cell activity drives gene expression, the user has control over what kinds of simulated behavior are in the data set. In that sense, defining the pathways is the first and most

important step of the analysis. When doing a parameter sensitivity, it would therefore make sense to analyze the sensitivity of a particular result to all other parameters while keeping the pathways constant.

CancerInSilico provides quite a bit of flexibility when defining the activity of a pathway. The activity is a function of the current time and a particular cell. The entire model state is available to query when calculating this activity. For instance, the default contact inhibition pathway has activity proportional to the local density of a cell. This is defined by examining a radius around the cell and calculating the percentage of the area that is occupied by other cells.

Statistical Error Model for Gene Expression Data

The pathways map the cellular growth model state to an activity measure in each gene between 0 and 1. There are two remaining steps that need to be done. First, the activity level needs to be translated to a mean expression level. This is done by calibrating all the pathways to some reference data set which gives the minimum and maximum expression levels for each gene. The pathway activity is mapped to this range with 0 corresponding to minimum expression and 1 corresponding to maximum expression. Then, depending on the simulated measurement platform, an error model is applied to the mean expression levels to get the final simulated gene expression data set. Here are all parameter involved in this stage of the simulation.

**General Error Model Parameters**		
**Parameter Name**	**Description**	**Value**
RNAseq	Generate RNA-seq data	boolean
singleCell	Generate single-cell data	boolean
nCells	Number of cells to sample at each time points	integer > 0
sampleFreq	How many hours between sample time points	integer > 0
nDummyGenes	Number of pure noise genes	integer > 0
dummyDist	Distribution for expression of pure noise genes	R function
combineFUN	How to combine expression values across pathways	R function

**Error Model Specific Parameters**		
**Parameter Name**	**Description**	**Value**
perError	Error for normal (microarray) model	double > 0
bcvCommon	Error for voom (RNA-seq) model	double > 0
bcvDF	Degrees for freedom for voom model	integer > 0
dropoutPresent	Simulate dropout in single-cell RNA-seq	boolean
dropoutMid	Parameter for dropout distribution	double
dropoutShape	Parameter for dropout distribution	double

The first set of parameters are general to the simulation of any type of data. **RNAseq** (as opposed to microrarray) and **singleCell** identify the measurement platform being simulated. **nCells** and **sampleFreq** determine how many cells are sampled and how often sampling is done. *CancerInSilico* allows an arbitrary number of dummy genes to be added to the simulation. These genes have expression sampled directly from some distribution and serve the purpose of adding pure noise to the data. **nDummyGenes** controls how many of these genes are present and **dummyDist** specifies their distribution. Finally, **combineFUN** determines how conflicting expression values for a single gene are resolved. This can happen when a single gene is annotated to more than one pathway. In this case, it will have multiple different mean expression levels from each pathway. This function is applied to all available mean expression levels and the result is used as the consensus of all the pathways. This is an important parameter since a simple average (default) of all expression levels implicitly assumes an independence across pathways. The user may need to adjust this function depending on the relationship between the pathways present in the simulation.

There are also parameters specific to a simulated measurement platform. When simulating log transformed microarray data, *CancerInSilico* uses a normal error model. The error has mean 0 and standard deviation of **perError**. When simulating bulk RNA-seq data the error model is adapted from the code for LIMMA voom with the parameters **bcvCommon** and **bcvDF** [3]. When simulating single cell RNA-seq data, the same error model from voom is used with the addition of the dropout model from Spatter [2]. There are three parameters that control this dropout distribution. All of these models are sensitive to parameter choosing and so it is recommended to run over a large range of values.

References

1. Drasdo D, Höhme S. Individual-based approaches to birth and death in avascular tumors. Math. Comput. Model. 2003; 37:1163–1175

2. Zappia L, Phipson B, Oshlack A. Splatter: simulation of single-cell RNA sequencing data. Genome Biol. 2017; 18:

3. Law CW, Chen Y, Shi W, et al. voom: precision weights unlock linear model analysis tools for RNA-seq read counts. Genome Biol. 2014; 15:R29

4. Chambers JM. Programming with Data: A Guide to the S Language. New York: Springer-Verlag. 1998;
